# Supplementary material for: Contractility-induced self-organization of smooth muscle cells: from multilayer cell sheets to dynamic three-dimensional clusters
Source: Commun Biol. 2023 Mar 11;6:262. doi: 10.1038/s42003-023-04578-8 (PMC10008632; doi:10.1038/s42003-023-04578-8)
Supplement: Supplementary file 3 — Description of Additional Supplementary Files [file 42003_2023_4578_MOESM3_ESM.pdf]

Description of Additional Supplementary Files

File name: Supplementary Movie 1

Description: Cluster formation: from cell sheet to three-dimensional structure

File name: Supplementary Movie 2

Description: Dynamics of opening of hole

File name: Supplementary Movie 3

Description: Aggregate development

File name: Supplementary Movie 4

Description: Aggregate tearing off and forming a cluster

File name: Supplementary Movie 5

Description: Elongated cluster rounding up

File name: Supplementary Movie 6

Description: Rounded cluster stabilization

File name: Supplementary Movie 7

Description: Cluster fusion

File name: Supplementary Movie 8

Description: Nucleation sites of hole opening

File name: Supplementary Movie 9

Description: Post-fusion rounding-up

File name: Supplementary Data

Description: The source data behind the graphs in the paper.
